# Single-cell transcriptomics reveals systemic immune dysregulation in non-segmental vitiligo

**DOI:** 10.3389/fimmu.2025.1698566

**Published:** 2025-12-08

**Authors:** Jialing Xiao, Xiaojuan Guo, Lingxue Gong, Qinhui Li, Kai Dong, Xiaoxin Guo, Huan Li, Ting Wang, Liang Wang, Weijia Wu, Chengzi Gan, Mingze Li, Bo Gong, Yixuan Jiang, Yixiao Wu, Yifan Hu, Liang Han, Jianing Yang, Yujie Mao

**Affiliations:** 1 Department of Human Disease Genes Key Laboratory of Sichuan Province and Institute of Laboratory Medicine, Sichuan Academy of Medical Sciences and Sichuan Provincial People’s Hospital, School of Medicine, University of Electronic Science and Technology of China, Chengdu, Sichuan, China; 2 Department of Dermatology, Sichuan Provincial People’s Hospital, School of Medicine, University of Electronic Science and Technology of China, Chengdu, China; 3 College of Clinical Medicine, Guizhou Medical University, Guiyang, China; 4 Medical Services Department, Sichuan Provincial People’s Hospital, School of Medicine, University of Electronic Science and Technology of China, Chengdu, Sichuan, China; 5 University of Electronic Science and Technology of China, School of Medicine, Chengdu, Sichuan, China

**Keywords:** GP-NSV, single-cell RNA sequencing, NK cell, KLRC2, Cytotoxic CD8⁺ T cell, FCGR3A, EGR1, B cell

## Abstract

**Background:**

Non-segmental vitiligo (NSV) is an autoimmune disorder characterized by irregular depigmented skin patches due to melanocyte loss, which causes considerable psychosocial burden. Although localized mechanisms underlying vitiligo pathogenesis have been studied extensively, investigations into peripheral blood mononuclear cells (PBMCs), key mediators of autoimmune diseases, remain limited.

**Methods:**

To address this gap, we performed single-cell RNA sequencing (scRNA-seq) on peripheral blood samples from 3 untreated patients with generalized, progressive non-segmental vitiligo (GP-NSV) and 3 healthy controls. Findings were validated using flow cytometry in an additional cohort of 7 GP-NSV patients and 30 controls. Computational analyses, including pseudotime trajectory reconstruction and pathway enrichment, were employed to characterize immune cell subsets and their functional states.

**Results:**

Vitiligo patients exhibited striking heterogeneity in PBMC subsets. KLRC2^+^ NK cells were markedly reduced and enriched in tumor necrosis factor (TNF) and apoptotic signaling pathways, a finding further confirmed by flow cytometry. Pseudotime analysis indicated that NK cells underwent negative regulation of DNA metabolic processes alongside activation of granzyme-mediated programmed cell death. In addition, the frequency of FCGR3A^+^ Cytotoxic CD8^+^T cell was reduced, with enrichment in T cell activation and differentiation signatures. STAM^+^ regulatory T cells (Tregs) were increased, whereas EGR1^+^ B cells were decreased, both subsets showing enrichment in pathways linked to osteoclast differentiation and calcium ion metabolism, suggesting a potential role of calcium homeostasis dysregulation in disease pathogenesis.

**Conclusions:**

This study provides the single-cell atlas of PBMCs in GP-NSV, uncovering profound transcriptional and compositional alterations across multiple immune cell subsets in active vitiligo. These findings offer novel insights into systemic immune dysregulation in GP-NSV and pave the way for novel targeted therapeutic strategies.

## 1 Introduction

Vitiligo is an autoimmune skin disease characterized by patches of white skin that can develop anywhere on the body and is caused mainly by CD8^+^ T cells attacking the epidermal melanocytes (1). On the basis of the rate of lesion expansion and its distribution characteristics, vitiligo can be categorized into two types: segmental vitiligo is characterized by unilateral and segmental lesion distribution, early onset, and rapid stabilization, whereas Non-segmental vitiligo (NSV) typically presents as bilateral, acral, or generalized symmetric lesions that evolve gradually over time (2). Notably, non-segmental vitiligo accounts for 84–95% of all vitiligo cases (3). It affects approximately 1% of the world’s population and have a huge psychosocial impact on people, such as shame, depression and anxiety (4) and are often associated with various systemic diseases including thyroid disease, rheumatoid arthritis, and alopecia areata (5). These research findings suggest that vitiligo may be a systemic immune disease. PBMCs serve as central sentinels of systemic immunity, acting both as frontline effectors of innate immunity and as bridges to adaptive immune responses. Through cytokine secretion, antigen presentation, and cytotoxic activity, they coordinate systemic immune reactions and can serve as sensitive indicators of disease progression and immune dysregulation in autoimmune conditions such as vitiligo. Nevertheless, their roles in vitiligo remain poorly understood.

Both lesional and peripheral immune dysregulation have been implicated in vitiligo. Cytotoxic CD8^+^ T cells, particularly those recognizing melanocyte-specific antigens, are considered key effectors mediating melanocyte destruction. These cells produce interferon-γ (IFN-γ), which drives chemokine production (CXCL9 and CXCL10) and recruits additional autoreactive T cells to the skin (6). NK cells and Innate Lymphoid Cell type 1(ILC1) also contribute to melanocyte injury through the IFN-γ–CXCR3B axis (7). Studies of peripheral blood using conventional immunophenotyping have revealed that vitiligo patients exhibit altered CD4^+^/CD8^+^ T cell ratios, expansion of activated T cell subsets, and changes in NK cell frequencies, reflecting systemic immune dysregulation associated with the disease (8). In addition, multiple studies have reported a reduction in both the frequency and suppressive function of Tregs in vitiligo patients, accompanied by decreased expression of FOXP3, IL−10, and TGF−β in the skin and peripheral blood, particularly during active disease, further underscoring the presence of systemic immune dysregulation (9). Emerging evidence suggests that peripheral immune subsets like B cells, γδ T cells, and tissue-resident-like T cells may play a role in immunity, but their contribution to vitiligo remains largely unexplored.

ScRNA-seq has revolutionized the ability to explore cellular heterogeneity at the individual cell level and uncover previously unrecognized immune cell subsets. It allows unbiased characterization of cell type composition and functional states, at single-cell resolution. Most scRNA-seq studies in vitiligo to date have focused on skin lesions, revealing tissue-resident memory T cells, melanocyte stress responses, and inflammatory signaling networks. For examples, regulatory T cells exert regulatory control over the development of vitiligo by modulating the interaction between the CCL5-CCR5 signaling pathways and CD8+ T cells, resulting in a trendic cycle (10). Simultaneously, Xu et al. (11) highlighted the distinctive role of epidermal fibroblasts in this process, as they secrete chemotactic factors to recruit and activate CD8+ cytotoxic T cells. Despite the current understanding that some cells play a role in the recruitment or activation of CD8+ cells, there remains a need for a more in-depth exploration of the functional characterization of other cells.

However, comprehensive profiling of the peripheral immune landscape in vitiligo remains limited, and the relationship between systemic immune states and cutaneous pathology is poorly defined.

In this study, PBMCs were isolated from three GP-NSV patients and three healthy controls for scRNA-seq to construct a high-resolution map of immune cell populations. We identified distinct immune cell subsets showing significant alterations in the patient group, including KLRC2^+^ NK cell and FCGR3A^+^ CD8^+^ Tem cells, explored their transcriptional programs and functional characteristics, and partially validated in subset by flow cytometry. Our findings provide new insights into systemic immune dysregulation and inform the development of targeted immunomodulatory therapies in vitiligo.

## 2 Methods and Materials

### 2.1 Ethics statement

This study was conducted in accordance with the Declaration of Helsinki. Ethical approval was obtained from the Institutional Review Board of Sichuan Provincial People’s Hospital (No. 2022731). Written informed consent was obtained from all participants prior to their inclusion in the study. The main research subjects were human peripheral blood samples. All personal data were anonymized to ensure participant confidentiality.

### 2.2 Subjects and clinical sample collection

In order to obtain data that better reflect the systemic immune status of vitiligo, we recruited patients with GP-NSV (12), who had depigmented areas covering more than 5% of body surface area and had the appearance of new lesions or extension of existing lesions within the last 3 months. Key exclusion criteria were previous use of any systemic treatment within 6 months. Healthy controls were recruited based on the absence of personal history of vitiligo, psoriasis, or any other autoimmune diseases. Additionally, all controls were in good general health with no significant comorbidities, including no history of systemic diseases or malignancy. The demographic characteristics of patients and healthy controls are detailed in **Supplementary Table 1**.

For single-cell sequencing, peripheral blood samples (10 mL/donor) were collected from three GP-NSV patients and three healthy controls using EDTA anticoagulant tubes (BD Vacutainer). For PBMC isolation, whole blood was diluted 1:1 with phosphate-buffered saline (PBS, Biosharp, Cat. No. BL302A) then layered over lymphocyte separation solution (Ficoll-Paque PREMIUM, Cat. No. 17544203) in 50 mL centrifuge tubes. Centrifugation was performed at 2000 rpm for 20 minutes at 20°C with brake disabled in a horizontal rotor. The intermediate white layer (peripheral blood mononuclear cells, PBMCs) was carefully aspirated and washed twice with PBS (300 × g, 10min), followed by resuspension in RPMI 1640 medium containing 0.04% BSA (Corning, Cat. No. 10-040-CVR). Cell concentration and viability were assessed using a Luna cell counter or Trypan blue staining.

For flow cytometry validation, peripheral blood samples from a larger cohort of 30 healthy controls and 7 GP-NSV patients were collected. Each sample consisted of 5 mL of peripheral blood. To maintain sample integrity, the collected blood samples were kept at room temperature and analyzed within 24 hours.

### 2.3 10x Genomics single-cell sample processing and cDNA library preparation

The freshly prepared single-cell suspension was adjusted to a concentration of 700–1200 cells/μl. According to the 10× Genomics Chromium Next GEM Single Cell 3′ Reagent Kit v3.1 User Guide (Cat. No. 1000268), the suspension was processed for loading and library construction. The constructed libraries were subjected to high-throughput sequencing on the Illumina NovaSeq 6000 platform using the PE150 platform. The raw data generated from high-throughput sequencing were in fastq format. These data were processed using the official 10x Genomics software, CellRanger, for quality statistics and alignment to the reference genome. The software quantifies high-throughput single-cell transcriptome data by recognizing cell-specific barcodes and unique molecular identifiers (UMIs) for different mRNA molecules within each cell. Data on cell viability, capture efficiency, and sequencing depth for each sample are provided in **Supplementary Table S1**.

### 2.4 Single-cell data preprocessing, gene expression quantification and cell-type determination

Based on the initial quality control from CellRanger, further quality control was performed using the Scrublet software package (13). Based on the UMI count matrix obtained from CellRanger, doublets were first identified and excluded using the Scrublet software. The matrix was then imported into Seurat (version 3.2.3) for subsequent quality control. The following filtering criteria were applied: (1) cells with a mitochondrial gene ratio exceeding 20%; (2) cells with fewer than 500 detected genes or a total UMI count below 1,000; and (3) after the above steps, cells with total UMI counts deviating beyond two standard deviations (mean ± 2 SD) from the overall mean were further removed to eliminate extreme outliers. To correct for batch effects across samples, canonical correlation analysis (CCA) in Seurat was employed for data integration. Prior to integration, the expression matrix of each sample was normalized using the NormalizeData function with the parameter normalization.method = “LogNormalize” and scale.factor = 10000. The top 2,000 highly variable genes were selected via the FindVariableFeatures function for downstream analysis. Subsequently, based on the first 10 principal components, cell clustering was performed using the FindNeighbors and FindClusters functions with a resolution of 0.8. Dimensionality reduction and visualization were achieved using Uniform Manifold Approximation and Projection (UMAP). Finally, the FindAllMarkers function was utilized to identify significantly upregulated marker genes in each cell cluster, and automated cell type annotation was performed using the SingleR package with reference datasets to minimize subjective bias. Differential expression analysis between groups was performed using the FindMarkers function in Seurat, with the parameters set to min.pct = 0.1 and test.use = “wilcox”. The identified marker genes were visualized using the VlnPlot and FeaturePlot functions. Using the SingleR package (14), cell type identification was performed on the basis of a single-cell reference expression quantitative public dataset. The expression profile of the cells to be identified was correlated with the reference dataset, and the cell type with the highest correlation in the reference dataset was assigned to the cells being identified. This approach reduces human subjective interference to some extent. The identification principle involves calculating the Spearman correlation between the expression profile of each cell in the sample and each annotated cell in the reference dataset. The cell type with the highest correlation to the sample cell’s expression in the reference dataset is selected as the final identified cell type.

### 2.5 Pseudotemporal trajectory analysis

Pseudotemporal trajectory analysis was performed on the target cell subpopulations using Monocle2 (15). Briefly, the UMI expression matrix and corresponding metadata from the Seurat object were converted into a CellDataSet object, with the expression family specified as the negative binomial distribution (negbinomial.size()). Size factors and dispersion values were then estimated using the estimateSizeFactors and estimateDispersions functions, respectively. Highly variable genes across cells were selected via the dispersionTable function for downstream ordering analysis. The data were subsequently reduced to two dimensions using the reduceDimension function, and cells were ordered along the inferred trajectory with the orderCells function. Finally, trajectory topology, dynamic gene expression patterns, and pseudotemporal clusters were visualized using the plot_cell_trajectory, plot_genes_in_pseudotime, and plot_pseudotime_heatmap functions, respectively.

### 2.6 KEGG and GO pathway analysis

Gene Set Preparation: Background gene set files were downloaded and organized from the KEGG database (https://www.kegg.jp/) and the GO database using the GSEABase package (v1.44.0). Pathway Activity Scoring: The GSVA package (v1.30.0) was used to calculate pathway activity scores for single cells. GSVA (Gene Set Variation Analysis) is an unsupervised gene set enrichment analysis method that calculates gene set enrichment scores for individual samples on the basis of gene set variation analysis. Differential Pathway Analysis: The LIMMA (16) package (v3.38.3) was used to calculate differences in pathway activity between different groups. LIMMA (Linear Models for Microarray Data) is a linear model method used to analyze differential expression in high-throughput gene expression data.

### 2.7 RNA extraction and library construction

Total RNA was extracted using the TRIzol reagent according to the manufacturer’s instructions. RNA purity and quantification were assessed using a NanoDrop 2000 spectrophotometer (Thermo Scientific, USA), and RNA integrity was evaluated with an Agilent 2100 Bioanalyzer (Agilent Technologies, Santa Clara, CA, USA). Transcriptome libraries were constructed using the VAHTS Universal V6 RNA-seq Library Prep Kit following the manufacturer’s protocol.

### 2.8 RNA sequencing and differential gene expression analysis

Library sequencing was performed on the Illumina NovaSeq 6000 platform, generating 150 bp paired-end reads. Each sample yielded approximately 50 million raw reads. Raw reads in FASTQ format were processed using fastp (17) to remove low-quality reads, obtaining clean reads for subsequent analysis. Read alignment to the reference genome was conducted using HISAT2 (18), followed by gene expression quantification in FPKM (19). The read counts for each gene were obtained using HTSeq-count (20).Principal component analysis (PCA) and visualization were performed using R (v3.2.0) to evaluate the biological reproducibility of the samples.

Differential gene expression analysis was conducted using DESeq2 (21), where genes with a q-value<0.05 and a fold change > 2 or<0.5 were defined as differentially expressed genes (DEGs). Hierarchical clustering analysis of DEGs was performed using R (v3.2.0) to illustrate gene expression patterns across different groups and samples. Additionally, radar plots were generated for the top 30 DEGs using the R package ggradar to visualize the expression changes of upregulated and downregulated genes.

### 2.9 Flow cytometry validation of KLRC2+ subset

Fresh heparin-anticoagulated whole blood samples were inverted 10 times at room temperature to mix thoroughly. Then, 5 μL of antibody and 25 μL aliquots of blood were added to a flow cytometry tube, followed by the addition of 1 μL KLCR2 antibody (BD, Cat# 748169) and 4 μL of a multi-color peripheral blood flow cytometry detection antibody kit (CD3/CD16 + 56/CD45/CD19, labeled with FITC/PE/PerCP/APC; Mindray, REF#105-004448-00). After vortex mixing, the samples were incubated in the dark for 15 minutes. Subsequently, 450 μL of red blood cell lysis buffer was added, vortexed, and incubated at room temperature for 5 minutes. Then, 2 mL of physiological saline was added, vortexed, and the samples were centrifuged at 450 g for 5 minutes. The supernatant was discarded, and 500 μL of physiological saline was added and stored. Finally, the samples were analyzed using the BD FACSCanto™ II flow cytometer.

### 2.10 Statistical analysis

FlowJo (version 10) was used to gate different cell subsets expressing KLRC2 in PBMCs, and the percentages of positive cells within each subset were exported. The data were then imported into SPSS (version 27) for statistical analysis. Based on the data distribution, the independent-samples Mann-Whitney U test was employed for comparative analysis, with a two-tailed *P*-value<0.05 considered statistically significant. GraphPad Prism (version 8.0.2) was used to generate bar graphs.

## 3 Results

### 3.1 scRNA-seq reveals five cell types in PMBC

**Figure 1:**
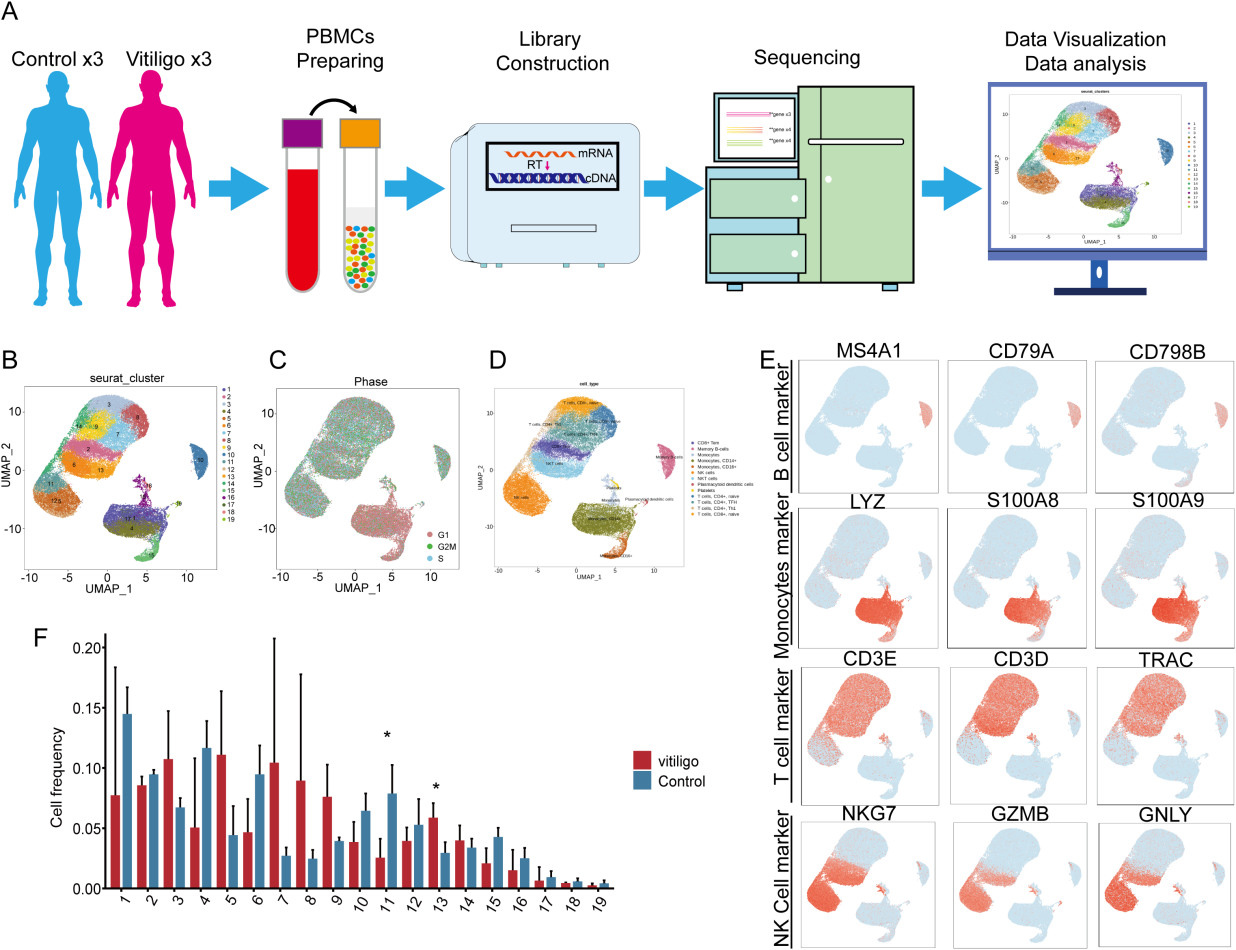
Single-cell transcriptomic analysis of peripheral blood mononuclear cells (PBMCs) from healthy controls and non-segmental vitiligo patients. **(A)** Schematic of the single-cell transcriptome sequencing workflow for the healthy control and patient groups. **(B)** UMAP plot showing the distribution of all cells, divided into 19 subclusters by clustering. After quality control, a total of 59,700 cells were obtained. **(C)** Cell cycle analysis of the total cell population. **(D)** Identification of cell types in the UMAP plot using Seurat and cell markers, including monocytes, T cells, B cells, and NK cells. **(E)** In each UMAP plot, classical marker genes were used to identify cell types. **(F)** Comparison of cell frequencies across different cell subclusters between the healthy control and patient groups. Data are presented as mean ± SEM, and paired t-test was used. *P <0.05.

The workflow for the single-cell sequencing study in this section encompasses clinical sample collection, isolation and preparation of PBMCs, library construction, sequencing, and subsequent data visualization and interpretation of biological significance (**Figure 1A**). We initially isolated 59,700 cells from PBMCs obtained from six samples for single-cell sequencing. These samples comprised three cases of patients with generalized, progressive non-segmental vitiligo (28,799 cells) and three healthy control cases (30,921 cells). Following cell collection, rigorous quality control procedures were applied, including the removal of doublets, empty droplets, and low-quality cells, before proceeding to subsequent analysis. Unsupervised clustering using Seurat revealed a total of 19 clusters (**Figure 1B**). The cell groupings for each subject are shown in (**Supplementary Figure 1A**). Cell cycle analysis revealed that the cell cluster in the lower-right corner was predominantly in the G1 phase (**Figure 1C**). To impart biological significance to these subpopulations, we first identified four major cell types on the basis of classical cell markers: T cells (CD3E, CD3D, TRAC), B cells (MS4A1, CD79A, and CD9B), NK cells (NKG7, GZMB, and IFNG), and monocytes (LYZ, S100AB, and S100A9) (**Figures 1D, E**). Notably, Clusters 6 and 13 exhibited expression profiles encompassing both T cell markers (CD3D, CD3E, and TRAC) and NK cell markers (NKG7 and GZMB) (**Figure 1E**). Considering this dual expression pattern, we classified these two cell subpopulations as NKT cells. In the initial identification of 19 cellular subtypes, we observed a decrease in Cluster 11 at the NK cell (*P*<0.05) and an increase in Cluster 13 at the NKT cell (*P*<0.05) within the vitiligo group (**Figure 1F**), suggesting a potential association with the onset and progression of vitiligo.

### 3.2 Heterogeneity in NK and NKT cells in vitiligo patients

**Figure 2:**
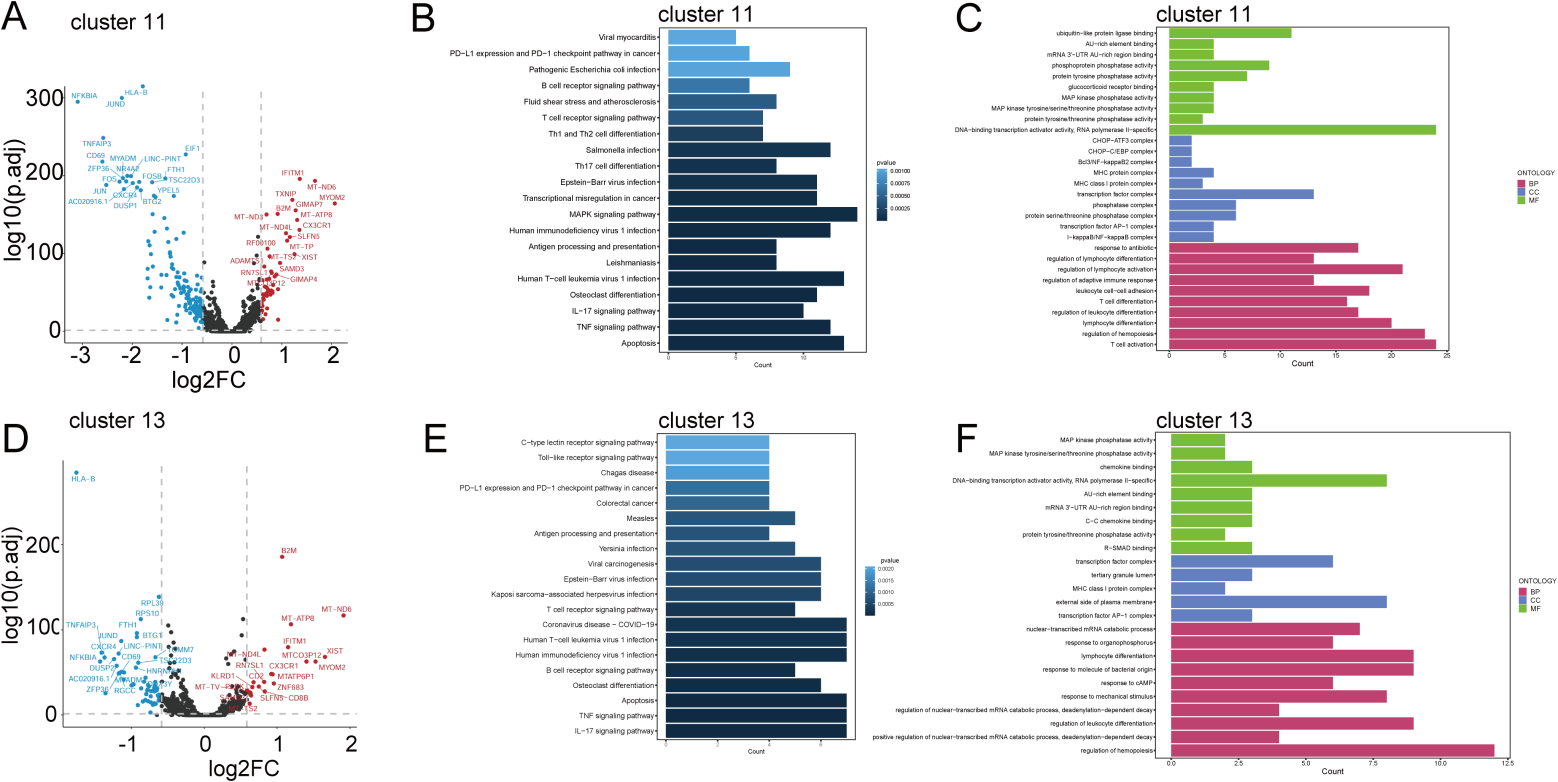
Gene expression and functional enrichment differences analysis of primary cell subsets with spectral differences in vitiligo and health control. **(A)** and **(D)** represent volcano plots of differentially expressed genes in Cluster 11 and Cluster 13 between normal controls and vitiligo patients. **(B)** and **(E)** show the KEGG functional enrichment analysis for Cluster 11 and Cluster 13, with the x-axis representing gene counts and the y-axis representing the relative functional categories of gene enrichment. **(C)** and **(F)** represent GO analysis for Cluster 11 and Cluster 13, with the red box indicating biological processes (BP), blue representing cellular components (CC), and green representing molecular functions (MF).

To delve deeper into the roles of NK and NKT cellular subtypes in the occurrence and development of vitiligo, we conducted differential gene expression analysis. The NK and NKT cell groupings for each subject are shown in (**Supplementary Figure 1B**). The results revealed that NK Cluster exhibited elevated expression of genes such as MYOM2, IFITM1, CX3CR1, GIMAP7, XIST, TXNIP, and SLFN5 (log2FC>1; *P*<0.05), whereas genes such as EGR1, KDM6B, DUSP2, IFNG, HLA-B, BTG2, FOSB, DUSP1, NR4A2, ZFP36, CXCR4, MYADM, JUND, FOS, JUN, TNFAIP3, CD69, and NFKBIA were downregulated (log2FC<-1; *P*<0.05) (**Figure 2A**). Further KEGG and GO analyses revealed enrichment in apoptotic signaling pathways in Cluster 11, explaining the observed reduction in NK cell numbers in vitiligo patients compared with those in the control group. Additionally, this cellular subtype was enriched in inflammatory pathways such as TNF signaling, IL-17 signaling, MAPK signaling, antigen processing and presentation, and Th17 cell differentiation (specific pathways to be investigated). GO analysis further revealed significant enrichment in T cell activation for this cellular subtype, suggesting a potential relevance to the occurrence of vitiligo (**Figures 2B, C**).

In contrast, NKT Cluster exhibited a significant increase in cell number compared with the normal control group. Differential gene expression analysis identified upregulated genes, including B2M, IFITM1, MT-ATP8, MTCO3P12, and MYOM2 (log2FC>1; *P*<0.05), whereas genes such as HLA-B, NFKBIA, TNFAIP3, CXCR4, FOS, DUSP2, AC020916.1, LINC-PINT, ZFP36, RGCC, JUND, CD69, NR4A2, and MYADM were downregulated (log2FC<-1; *P*<0.05) (**Figure 2D**). Through combined KEGG and GO analyses, we found that Cluster 13 was associated with biological processes such as the IL-17 signaling pathway, TNF signaling pathway, B cell receptor signaling pathway, T cell receptor signaling pathway, and hematopoietic regulation (**Figures 2E, F**). This may explain the observed increase in cell numbers and suggest a regulatory role in the immune system.

### 3.3 The reduction of the NK cell subset containing KLRC2

**Figure 3:**
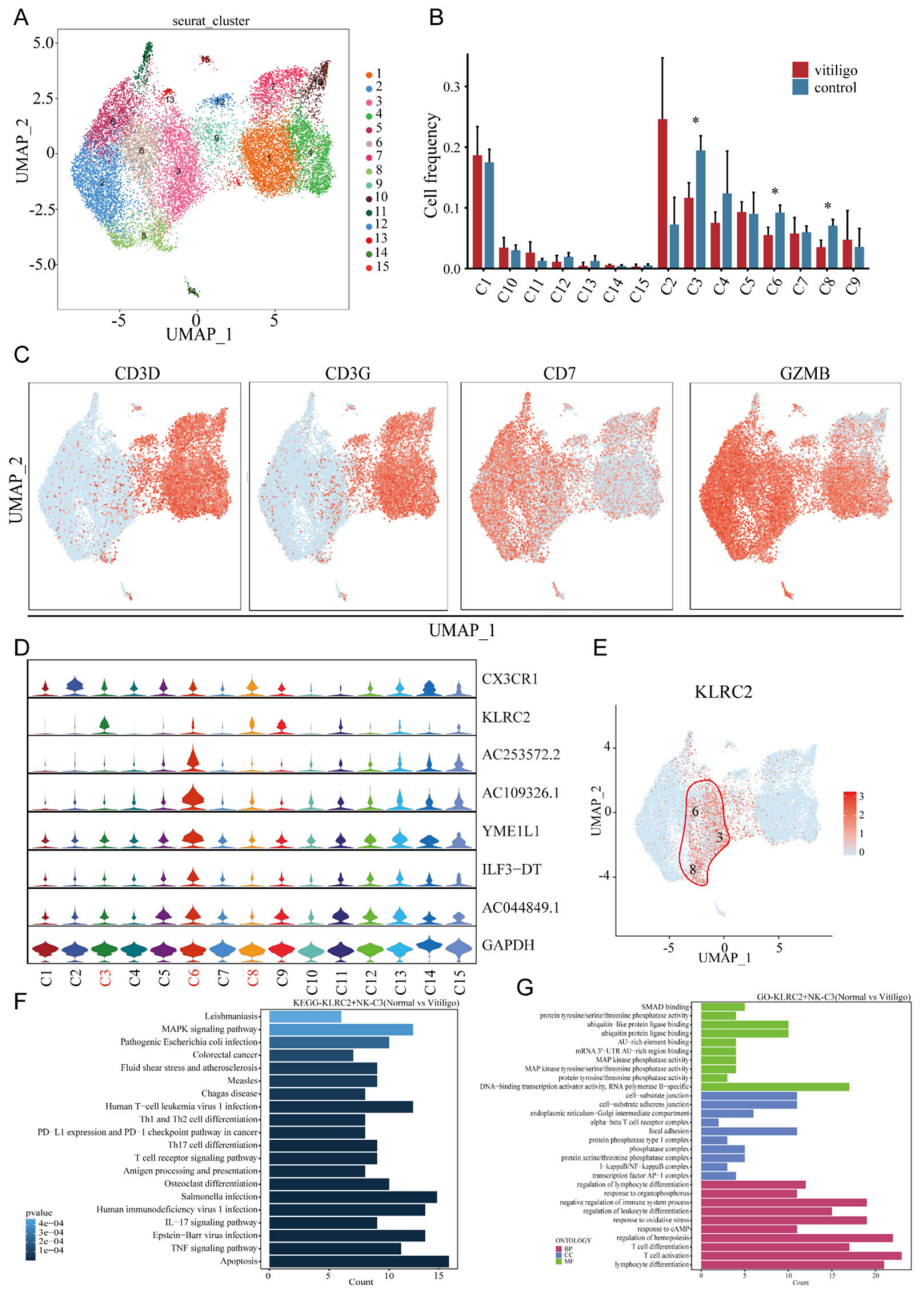
Transcriptional profiling of NK cell subpopulations in non-segmental vitiligo and healthy controls. **(A)** UMAP plot showing the reclustering of NK cell subpopulations into 19 distinct clusters, with each cluster color-coded and numbered. **(B)** Cell frequency analysis of NK cell subpopulations in healthy controls and non-segmental vitiligo patients, highlighting differences in clusters 3, 6, and 8 between the groups. **(C)** Further classification of NK cell subpopulations using classical cell markers to identify NK cells. **(D)** Violin plots identifying specific differentially expressed genes within NK cell subpopulations. **(E)** UMAP plot illustrating the specific expression of KLRC2 in clusters 3, 6, and 8. **(F)** KEGG pathway enrichment analysis of KLRC2^+^ NK cells. The x-axis represents the enrichment score, and the y-axis lists the pathways. Pathways are ordered by significance, with the most significantly enriched pathways at the top. **(G)** GO enrichment analysis of DEGs in KLRC2^+^ NK cells, highlighting key categories: Biological Process (BP), Cellular Component (CC), and Molecular Function (MF). The x-axis represents enrichment scores or gene counts, and the y-axis lists the GO terms. Terms are ranked by significance, with the most significant terms at the top. Data are presented as mean ± SEM, and paired t-test was used. *P<0.05.

To identify the specific NK and NKT cell subpopulations potentially involved in vitiligo, we extracted 9,257 cells from all NT and NKT cluster and re-clustered them into 15 distinct subtypes (**Figure 3A**). Notably, we observed a decrease in the prevalence of several cellular subtypes, namely Clusters 3, 6, and 8, in vitiligo patients (**Figure 3B**). We analyzed the expressions of CD3D, CD3E, CD7, and GZMB to determine whether these cells exhibit characteristics of NK cells and NKT cells (**Figure 3C**). In the quest for common markers among these cellular subtypes, we identified the gene marker KRLC2 through violin plots (**Figures 3D, E**), which was expressed consistently across Clusters 3, 6, and 8. Although the specificity of this cellular marker is not high, it currently serves as a relatively robust marker for labeling these subtypes. Consequently, we defined this cellular subtype as KLRC2^+^ NK cells. This cell groupings for each subject are shown in (**Supplementary Figure 2A**). In this clusters, certain genes, such as MYOM2, XIST, CX3CR1, and IFITM1, were totally significantly upregulated (log2FC>1; *P*<0.05), whereas others, including BTG2, FOS, CXCR4, CD69, TNFAIP3, and NFKBIA, were significantly downregulated (log2FC<-1; *P*<0.05). Further KEGG and GO analyses revealed significant enrichment of T cell activation, IL-17 signaling, apoptosis, and oxidative stress response pathways in Clusters 3 and 6 (**Figures 3F, G**).

To further validate the reduction of KLRC2^+^ NK cell subsets, we collected samples from 7 vitiligo patients of different age groups and 30 healthy controls (**Supplementary Table 2**). The frequency of KLRC2^+^ NK cells was assessed using flow cytometry, with the results shown in (**Supplementary Figures 3A, B**). Interestingly, flow cytometry data revealed a significant reduction in KLRC2 expression on the surface of T cells, B cells, and monocytes in the patient cohort, whereas the frequency of KLRC2^+^ NK cells showed no significant difference compared with healthy controls. Consistent with this, we performed bulk RNA sequencing on samples corresponding to those used in single-cell sequencing, and the results showed a significant decrease in KLRC2 expression (**Supplementary Figures 3C, D**). Consistent with vitiligo, a reduction in the CD56^+^ NK cell subpopulation was also observed in other autoimmune diseases, including hyperthyroidism and alopecia areata (22, 23).

### 3.4 NK cell subsets monocle analysis to delve its lifespan progression in vitiligo

**Figure 4:**
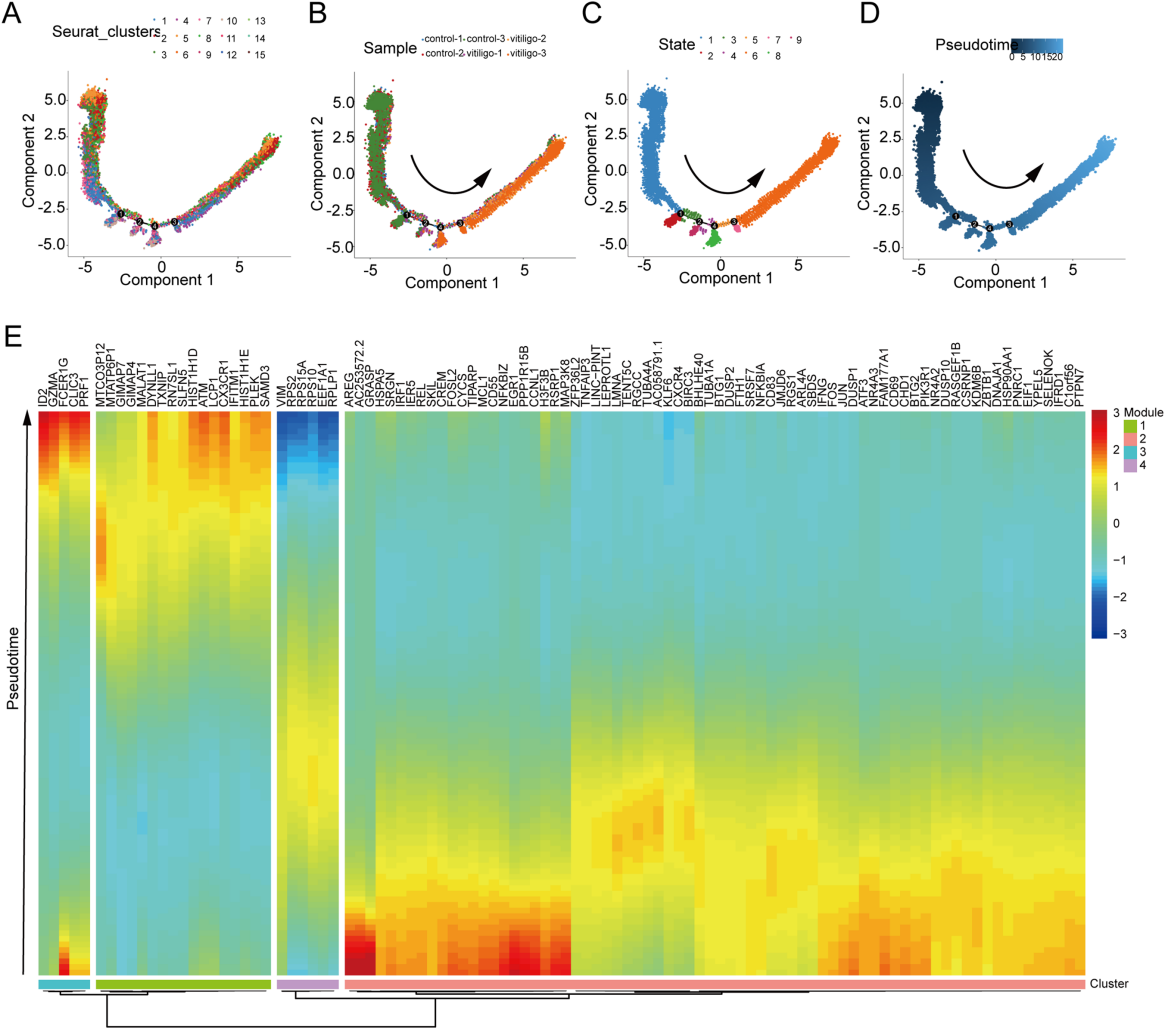
Pseudotime analysis reveals the developmental trajectories of NKT cell subpopulations. **(A)** Pseudotime trajectory plot of different NKT cell subpopulations showing developmental paths. Distinct branches represent different trajectories of cell development. **(B)** Pseudotime trajectory plot based on individual samples from healthy controls and non-segmental vitiligo patients, highlighting developmental paths and branching patterns. **(C)** Trajectory plot showing NKT cell developmental changes across different pseudo-time states. Various branches indicate different developmental trajectories. **(D)** Pseudo-time trajectory plot based on Pseudotime scoring, illustrating cell development along different paths. **(E)** Heatmap displaying the expression patterns of significantly varying genes in NKT cells on the basis of Pseudotime assessment, with emphasis on gene expression trends across different pseudo-time points.

The observed decrease in inhibitory receptors (CD158a^+^) and increase in activating receptors on NK cells have been implicated in the pathogenesis of vitiligo, suggesting a potential role for NK cell activation in disease development and progression (24). Similarly, we found that the NK cell subset KLRC2^+^ is reduced in non-segmental vitiligo patients, suggesting that different NK cell subsets may play distinct roles in disease development. Therefore, we used pseudotime analysis to investigate the branching of NK cell subsets to explore the impact of each subset on vitiligo. Pseudotime scores and sample distributions were used to determine the direction of NK cell differentiation (**Figures 4A–D**). Furthermore, we identified pseudotime-dependent genes across four distinct modules (modules 1–4), each characterized by unique expression patterns (**Figure 4E**). In the late stage of disease in NK cells, Module 1 and Module 3 exhibited high expression of genes such as GZMA, DYNLL1, and ATM, LCP1 which are associated with the negative regulation of DNA metabolic processes and the granzyme-mediated programmed cell death signaling pathway. In contrast, in the early stage of disease in NK cells, Module 2 and Module 4 showed high expression of genes such as HSPA5, RGCC, BIRC3, HSP90AA1, NFKBIZ, ZFP36L2, KLF6, and JMJD6, which are associated with programmed cell death and the positive regulation of macromolecule metabolic processes.

Taken together, these findings indicate that NK cell functions undergo distinct shifts during the course of vitiligo, transitioning from early-stage profiles in NKT cells enriched for genes linked to programmed cell death and macromolecule metabolism, to late-stage NK cell profiles characterized by genes associated with the suppression of DNA metabolic processes and granzyme-mediated cytotoxicity, suggesting a potential role for these functional changes in disease progression.

### 3.5 PBMC scRNA-seq reveals functional and phenotypic changes in Treg and FCGR3A^+^ CD8^+^ T cells in vitiligo patients

**Figure 5:**
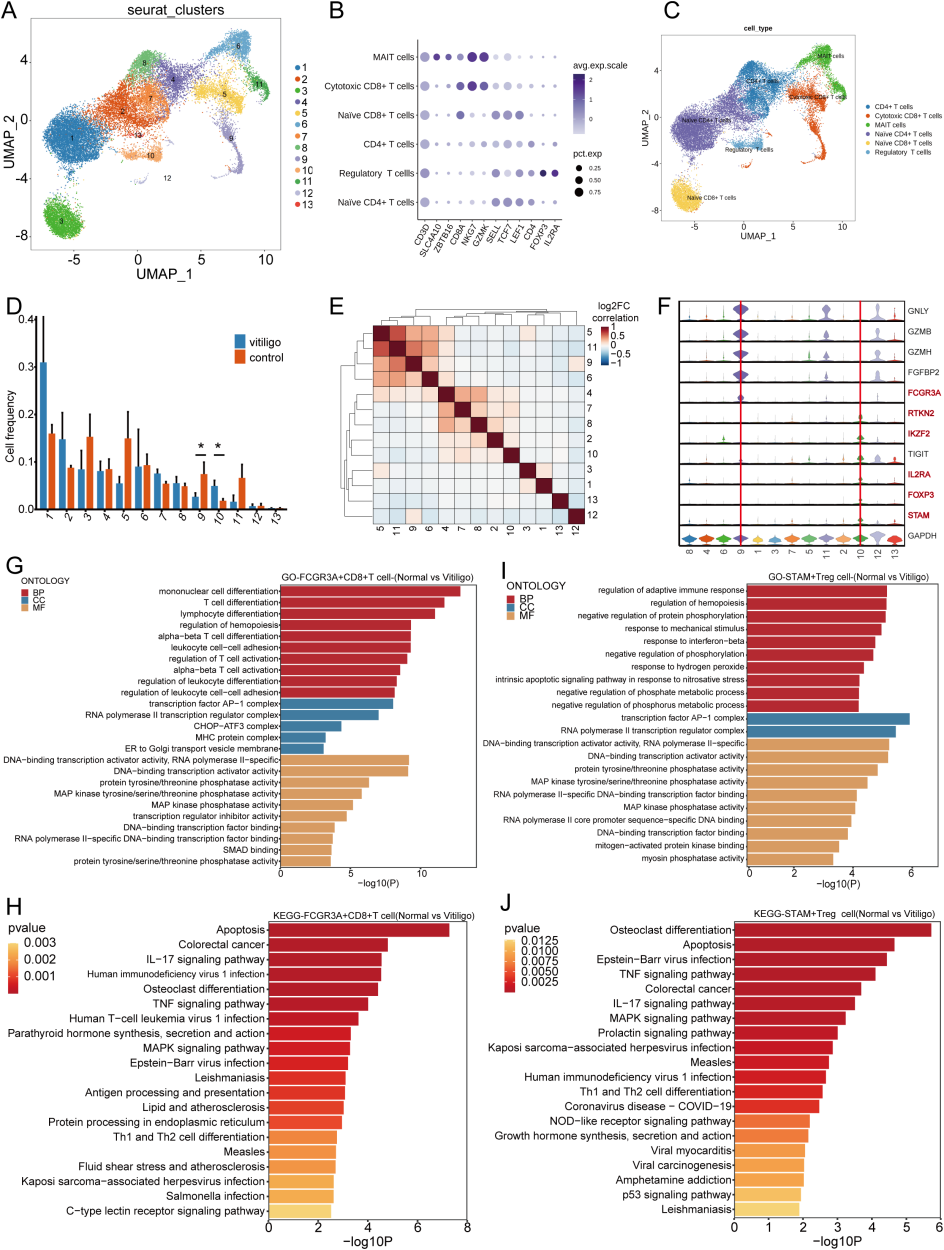
Re-clustering transcriptomic analysis of T cell subpopulations in healthy controls and non-segmental vitiligo patients. **(A)** UMAP plot showing the re-clustering of T cell subpopulations into 13 new clusters. **(B)** Dot plot displaying the marker genes used to delineate major immune cell subsets. **(C)** UMAP visualization illustrating the identification of T cell subsets. **(D)** Bar chart depicting cell frequencies across different groups. **(E)** Heatmap illustrating the correlation between T cell subpopulations, with colors indicating the strength of the correlation from negative (blue) to positive (red). **(F)** Violin plot showing the expression of specific genes in different T cell subpopulations. **(G)** GO pathway enrichment analysis for FCGR3A^+^ Cytotoxic CD8 ^+^ T cells, with the x-axis representing enrichment scores and the y-axis listing pathways, ordered by significance. **(H)** KEGG analysis of differential gene expression in FCGR3A^+^ Cytotoxic CD8 ^+^ T cells, showing enrichment in biological processes (BP), cellular components (CC), and molecular functions (MF). The x-axis represents enrichment scores or gene counts, and the y-axis lists GO terms, ordered by significance. **(I)** GO pathway enrichment analysis for STAM^+^Treg cells, with the x-axis representing enrichment scores and the y-axis listing pathways, ordered by significance. **(J)** KEGG analysis of differential gene expression in STAM^+^Treg cells, showing enrichment in BP, CC, and MF. The x-axis represents enrichment scores or gene counts, and the y-axis lists GO terms, ordered by significance. Data are presented as mean ± SEM, and paired t-test was used. *P<0.05.

T cell subsets play critical roles in the pathogenesis and progression of vitiligo. For example, CD8+ T cells primarily mediate melanocyte death (25). However, the functions of other T cell subsets remain unclear. Therefore, we reclustered the T cell subsets and identified 13 new subsets (**Figure 5A**). The T cell groupings for each subject are shown in (**Supplementary Figure 1C**). Using the SingleR package and classical cell markers (**Figure 5B**), we manually defined CD4+ T cell, Naïve CD4+ T, Naïve CD8+T, Cytotoxic CD8+T, MAIT T cell and Treg subsets (**Figure 5C**) (26). First, we conducted cell spectrum analysis on various subpopulations and found that the number of cells in Cluster 10 (**Figure 5D**), which contains Tregs and exclusively expresses FOXP3, had increased (P = 0.012) (**Figure 5F**). This finding is consistent with previous reports (27). We used a gene expression heatmap to identify some uniquely expressed marker genes in Treg cells, including RTKN2, IKZF2, TIGIT, IL2RA, CTLA4, and STAM. These genes may serve as markers for identifying Treg cells in vitiligo. The specific expression of STAM in Treg cells allowed us to define a novel STAM^+^ Treg subset in vitiligo, which may contribute to disease development. The STAM^+^Treg cell groupings for each subject are shown in (**Supplementary Figure 2C**). By examining the functions of these genes in Treg cells, we found that they are involved in multiple biological processes and pathways, including apoptosis, EBV infection, the TNF signaling pathway, and the IL-17 signaling pathway, as well as the negative regulation of protein phosphorylation, regulation of hematopoiesis, and modulation of the adaptive immune response, highlighting the immunoregulatory role of Treg cells (**Figures 5I, J**). Interestingly, Treg cells exhibit significant osteoclast differentiation activity, suggesting a potential link between vitiligo and osteoporosis (28). Similarly, by analyzing cell frequency differences and correlations, we found that the cell subpopulations in Cluster 5(P = 0.047) and Cluster 9(P = 0.034) were significantly reduced in patients (**Figures 5D, E**), and they identified as Cytotoxic CD8+T cells (**Figure 5C**). Violin plots indicated that FCGR3A may serve as a specific marker for this subpopulation (**Figure 5F**); accordingly, we defined FCGR3A^+^ Cytotoxic CD8^+^ T cells and observed a reduction of this subset in patients. This cell groupings for each subject are shown in (**Supplementary Figure 2B**). Functional enrichment analysis revealed that this cell subset is associated with biological processes related to mononuclear cell differentiation, T cell differentiation, and lymphocyte differentiation. These differentiation processes may contribute to the progression of vitiligo (**Figures 5G, H**).

### 3.6 Single-cell RNA sequencing of PBMCs unravels altered functional states in the EGR1^+^ naïve B cell compartment in vitiligo

**Figure 6:**
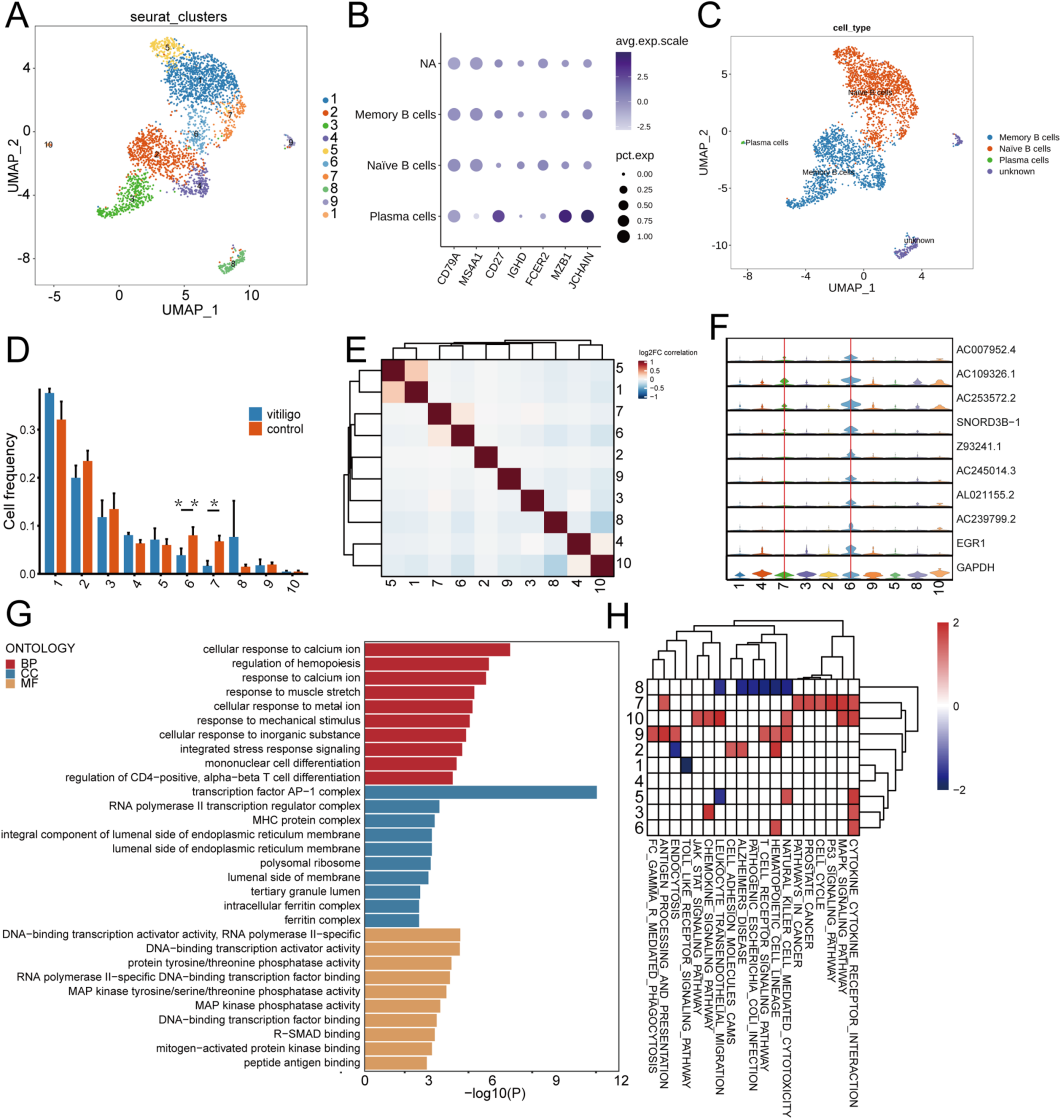
Re-clustering transcriptomic analysis of B cell subpopulations in healthy controls and non-segmental vitiligo patients. **(A)** UMAP plot showing B cell re-clustering into 10 new subpopulations, with cells marked by color and number. **(B)** Dot plot displaying the marker genes used to delineate B cell subsets. **(C)** UMAP visualization illustrating the identification of B cell subsets. **(D)** Bar chart of B cell frequencies across groups. **(E)** Correlation heatmap of B cell subpopulations, with colors indicating the strength of the correlation from negative (blue) to positive (red). **(F)** Violin plot of specific gene expression in B cell subpopulations. **(G)** KEGG pathway enrichment analysis for naive B cells, with x-axis showing enrichment scores and y-axis listing pathways, ordered by significance. **(H)** Gene Set Enrichment Analysis (GSEA) of B cell subsets in vitiligo patients. The x-axis represents enriched pathways, and the y-axis represents cell subsets.

The role of B cells in the development of vitiligo is not yet clear. However, B cell activating factors may activate self-reactive B cells to produce auto-antibodies against melanocytes. These auto-antibodies may function as cellular adjuvants, activating CD4+ T cells and thereby enhancing their helper effect on the activation of CD8+ T cells (29). To investigate the role of B cells in vitiligo, we re-clustered B cells into 10 new subpopulations (**Figure 6A**) and used singleRs and musical marker (**Figure 6B**) to manually define them as memory B cells, naive B cells and plasma (**Figure 6C**). The B cell groupings for each subject are shown in (**Supplementary Figure 1D**). We calculated the frequencies of these cells and found that Clusters 6(P = 0.001) and 7(P = 0.028) were reduced in vitiligo patients (**Figure 6D**). Correlation analysis revealed that these subpopulations were highly correlated (**Figure 6E**). Violin plots revealed that Cluster 6 specifically expressed EGR1, which regulates CD44 transcription in B cells, promoting B cell homing and migration; thus, we defined these cells as EGR1^+^ B cells (**Figure 6F**). This cell groupings for each subject are shown in (**Supplementary Figure 2D**). Functional studies indicated that memory B cells might be related to calcium ion cellular responses (**Figure 6G**). This is related to H_2_O_2_-mediated oxidative stress in the epidermis, leading to altered calcium binding and a significant decrease in calcium ATPase activity (30). Additionally, GSVA analysis indicated that EGR1+ memory B cells are enriched in cytokine-cytokine receptor interactions and hematopoietic cell lineage pathways (**Figure 6H**).

These findings suggest a functional role for these B cells in vitiligo; however, further research is needed to elucidate their exact mechanisms of action.

### 3.7 PBMC scRNA-seq identifies twelve monocyte subsets

**Figure 7:**
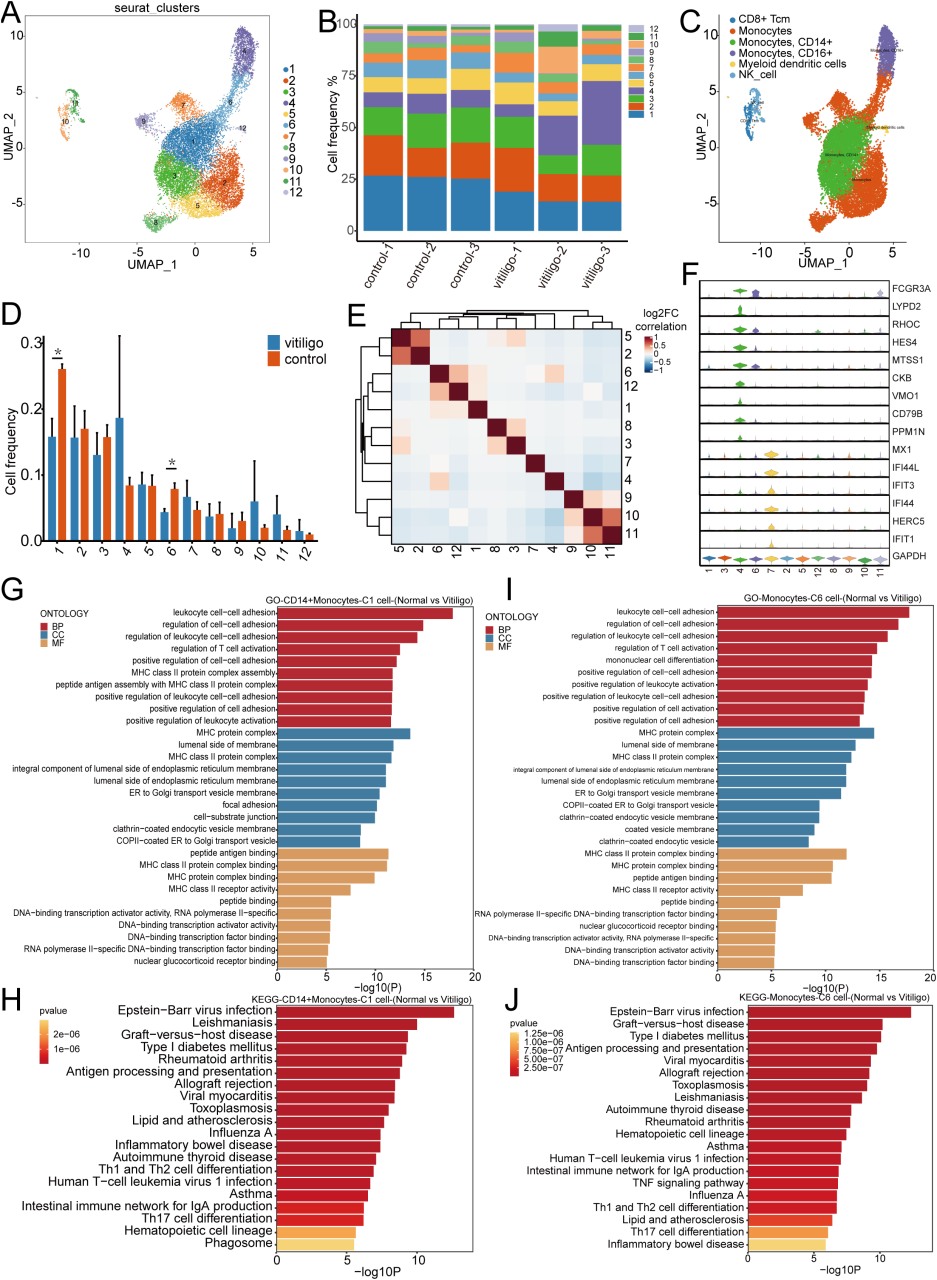
Transcriptomic analysis of monocyte subpopulations re-clustering in healthy controls and non-segmental vitiligo patients. **(A)** UMAP plot illustrating the re-clustering of monocyte subpopulations into 12 distinct clusters, with individual cells denoted by color and numerical labels. **(B)** Comparison of monocyte frequencies across samples from healthy controls and non-segmental vitiligo patients. **(C)** UMAP plot showing the automatic annotation of monocyte subpopulations and other cell types using SingleR. **(D)** Bar chart representing the distribution of monocyte frequencies between the healthy controls and non-segmental vitiligo groups. **(E)** Heatmap depicting the correlations between monocyte subpopulations, with color intensity indicating the strength of the correlation, ranging from negative (blue) to positive (red). **(F)** Violin plot displaying the expression patterns of specific genes across different monocyte subpopulations. **(G–J)** KEGG and GO enrichment analyses of CD14+ monocytes and cluster 6 monocytes, highlighting the functional enrichment and differential gene expression within biological processes (BP), cellular components (CC), and molecular functions (MF). The x-axis represents enrichment scores or gene counts, and the y-axis lists pathway or GO term names, ranked by significance.

Current research on the role of monocytes in the development of vitiligo is limited. Previous studies have indicated that monocytes accumulate a significant amount of hydrogen peroxide (31), leading to altered enzyme activity. These findings suggest that oxidative stress plays a crucial role in the pathogenesis of vitiligo. To further investigate the potential role of monocytes in the development and progression of vitiligo, we re-clustered the monocytes into 12 subpopulations (**Figures 7A, B**). Using classical cell markers and the SingleR method (14), we defined these subpopulations as monocytes, CD14+ monocytes, CD16+ monocytes (32–34), and myeloid dendritic cells (**Figure 7C**) (35). The monocyte cell groupings for each subject are shown in (**Supplementary Figure 1E**). We subsequently performed statistical analysis of the frequencies of the re-clustered cell subpopulations and found a significant difference between Cluster 1(P = 0.003) and Cluster 6 (P = 0.004) (**Figure 7D**). Correlation analysis further revealed a positive correlation between these two subpopulations (**Figure 7E**). We attempted to identify specific marker genes for these two subpopulations, but violin plot analysis did not reveal any significantly different marker genes within the clusters (**Figure 7F**). We then applied KEGG and GO analyses to explore their functional pathways (**Figures 7G–J**), which indicated that these two subpopulations are involved in pathways related to leukocyte cell-cell adhesion, the regulation of cell-cell adhesion, the regulation of leukocyte cell-cell adhesion, and the regulation of T cell activation. These pathways suggest that leukocyte cell-cell adhesion is a critical process involving multiple pathways and molecules, including integrins, selectins, ICAM, and VCAM, which play key roles in inflammation and immune system functions. Additionally, these processes are accompanied by T cell activation, indicating that monocytes may play a role in the pathogenesis of vitiligo. However, further studies are required to confirm this potential involvement.

## 4 Discussion

Vitiligo is a depigmenting skin disorder caused by the loss of melanocytes, affecting approximately 1% of the global population. The NSV accounts for 84–95% of cases and imposes a substantial psychological burden on patients. Beyond its cutaneous manifestations, vitiligo is frequently accompanied by systemic autoimmune diseases, underscoring its value as a model for studying systemic immune dysregulation. Current models posit that disease spread is primarily driven by the IFN-γ to JAK/STAT axis, leading to elevated CXCL9/CXCL10 expression and subsequent recruitment of CXCR3^+^ cytotoxic CD8^+^ T cells to new skin sites (36). However, the “selectivity” underlying long-range lesion dissemination remains poorly understood. To address this gap, we performed single-cell RNA sequencing of PBMCs, a compartment reflecting integrated systemic immune activity, to gain a new immunological perspective on vitiligo pathogenesis. Our analysis revealed dynamic alterations and functional roles of KLRC2^+^ NK cells, Treg cells, FCGR3A^+^ Cytotoxic CD8 ^+^ T cells, and EGR1^+^ B cells, opening new avenues for systemic immunology-oriented research in vitiligo.

The balance of immune cells plays a crucial role in the progression of vitiligo (37–39). The inconsistency in reported NK cell abundance in vitiligo patients across different studies may be attributed to variations in disease stages and individual patient differences (24, 40). Cytokines derived from NK cells and their cytotoxic functions regulate immune responses and may contribute to various immune-mediated diseases (41), such as ankylosing spondylitis, Behçet’s disease, and multiple sclerosis.

Here, we report a reduction in NK cells accompanied by an increase in NKT cells in vitiligo patients. Notably, the diminished NK cell population was characterized by a distinctive marker profile—KLRC2—allowing us to define this subset as KLRC2^+^ NK cells. Functional enrichment analysis indicated that this subset is associated with apoptosis, T cell activation, TNF signaling, and lymphocyte differentiation. Consistent with our single-cell data, the enrichment of apoptosis-related genes aligns with the observed decrease in KLRC2^+^ NK cell abundance. Moreover, bulk RNA-seq analysis of PBMCs also revealed a reduction of KLRC2 expression, whereas the roles of this subset in other pathways remain to be further elucidated. Although flow cytometric validation of KLRC2 expression in bulk NK cell showed no statistical significance, this discrepancy may stem from two methodological considerations: (1) Structural homology between KLRC2 and KLRC1 (differing by only two amino acids) may compromise antibody specificity, potentially masking true expression differences; (2) Upon re-clustering the original differential spectrum cell subsets, Cluster 11 and Cluster 13, we further identified 15 clusters. Using canonical lineage markers (NK cells: CD7, GZMB; T cells: CD3D, CD3G), three intermediate clusters (Clusters 3, 6, 8) were identified at the NK-T cell interface, suggesting a transitional immunophenotype. Cross-compartment analysis demonstrated no significant KLRC2 modulation in sorted NK/NKT populations, but revealed marked downregulation in T cells from patients. This phenomenon may be due to Clusters 3, 6, and 8 being in a transitional state between NK and NKT cells, representing a T cell subset that was not distinctly classified at the single-cell level but became evident through flow cytometry analysis. This result aligns with single-cell sequencing data, further supporting the existence of a transitional T cell subset between NK and NKT cells. Moreover, the decreased KLRC2 expression in this T cell subset in patients suggests its potential as a diagnostic marker for vitiligo, highlighting its relevance in disease pathogenesis and biomarker development. Aberrant expression of KLRC2 in NK cells is critical for maintaining the microenvironment essential for tumor initiation and progression (42). Its absence also plays a pivotal role in immune regulation during transplantation (43)or infection (44). Upregulation of KLRC2 in vitiligo skin lesions has been reported, suggesting its potential involvement in local immune responses (45). However, the fate, function, and underlying mechanisms of the activated NK cell subset represented by KLRC2 in peripheral blood remain largely unexplored. Here, we demonstrate for the first time that KLRC2^+^ NK cells are markedly reduced in vitiligo patients and exhibit features enriched for apoptosis-related and immunoregulatory pathways. These findings not only fill a critical gap in the current literature but also provide new evidence and insights for understanding the pathogenesis of vitiligo from a systemic immunological perspective.

Disease progression is a dynamic process. To explore the transcriptional alterations in NK and NKT cells during this process, we investigated this issue using pseudotime analysis. In the early stage of the disease, NK cells exhibited high expression of genes such as GZMA (46), DYNLL1, ATM, and LCP1, which are involved in the negative regulation of DNA metabolism and granzyme-mediated programmed cell death. These findings are consistent with existing studies and suggest that NK cells in early-stage vitiligo may eliminate aberrant melanocytes through granzyme-mediated cytotoxicity while simultaneously limiting their own DNA metabolic activity to maintain cellular homeostasis. In the late stage of the disease, NK cells showed elevated expression of HSPA5, RGCC, BIRC3, HSP90AA1, NFKBIZ, ZFP36L2, KLF6, and JMJD6, genes associated with programmed cell death and the positive regulation of macromolecule metabolism. The expression patterns of these genes align with previously reported NK cell functional characteristics, indicating that in late-stage vitiligo, NK cells may adapt to chronic inflammatory conditions or sustained tissue damage by modulating metabolic and stress-response pathways. Future studies should focus on elucidating the activation mechanisms of NK cells, the molecular basis underlying their functional transitions, and their specific roles in disease progression, with the aim of informing strategies for early diagnosis and targeted therapy in vitiligo.

In vitiligo, the infiltration of CD8+ T cells is mediated primarily by the secretion of chemokines such as IFN-γ by dermal cells (11, 47, 48). These chemokines recruit and activate CD8+ T cells, which then infiltrate the dermal tissue and target melanocytes. Treg cells, through the CCL5-CCR5 axis, exert a protective effect on melanocytes by modulating the activity of CD8+ T cells (10). Existing evidence indicates that a subset of tissue-resident memory T (TRM) cells patrols the epidermis and papillary dermis, and are reactivated by homologous antigens, leading to relapse (49). Although the localized pathogenesis and recurrence of vitiligo have been extensively studied, there is still a lack of research on immune cell subsets in the context of vitiligo spread (50). Investigating the characteristics of immune cells in peripheral blood of progressive patients may be a crucial step in addressing the issue of vitiligo expansion. Here, we identified 13 cell subsets and found that, compared with those in healthy individuals, Treg cells in progressive vitiligo patients are more abundant and express genes such as RTKN2, IKZF2, IL2RA, and STAM. STAM is a cytosolic signal-transducing adaptor molecule that regulates JAK-STAT signaling through its role in cytokine receptor transport and serves as a critical negative regulator of the cGAS-STING pathway, thereby restraining hyperactivation of the innate immune response (51, 52).In addition to STAM, these genes are critical in autoimmune diseases and Treg activation (53–55). Notably, the STAM gene has not been previously reported in Treg cells, suggesting that it may play a significant role in Treg regulation in vitiligo. There, we defined this cell subset as the STAM^+^ Treg cell subset.

During the pathogenesis of vitiligo, regulatory T cells (Tregs) recognize the CCL5 ligand secreted by CD8^+^ T cells through their own surface CCR5 receptor, thereby exerting immunosuppressive functions and restraining the overactivation of CD8^+^ T cells (10). However, despite the increased abundance of Tregs in both the epidermis and peripheral blood of patients, they fail to effectively curb disease progression, suggesting that Tregs may reside in a “functionally impaired” state (56, 57), characterized by Th1-Treg polarization (58). Our single-cell transcriptomic analysis revealed a significant upregulation of the transcription factor STAT1 in patient-derived Tregs (56, 57), along with activation of the tumor necrosis factor (TNF)-related signaling pathway, further corroborating the shift of Tregs toward a pro-inflammatory Th1 phenotype (59). Notably, STAM, functioning as an intracellular signal-transducing adaptor molecule, may facilitate STAT1 activation via modulation of the JAK-STAT pathway. Collectively, these alterations lead to diminished immunosuppressive capacity and enhanced pro-inflammatory properties of Tregs, thereby impairing their regulatory control over CD8^+^ effector T cells and ultimately exacerbating the initiation and progression of vitiligo.

The secretion of cytokines may promote osteoporosis and is associated with skin diseases, suggesting an immune-mediated connection between them (28, 60). The role of CD8+ T cells as the primary cell population that targets melanocytes has not yet been fully elucidated in peripheral blood. Our data revealed a significant reduction in the FCGR3A^+^ Cytotoxic CD8 ^+^ T cell subset in patients. Functional enrichment analysis also indicated that this cell subset is associated with apoptosis-related signaling pathways, corroborating the reduction in cell abundance observed in patients. Additionally, the cells expressed pathways related to T cell differentiation and activation, suggesting an abnormal activation state of immune cells. FCGR3A, which is commonly associated with NK cells, mediates antibody-dependent cellular cytotoxicity (ADCC), enabling T cells to perform ADCC functions (61, 62). But, for the CD8+ T cell subset expressing FCGR3A, previous study (63)has shown that it is a terminally differentiated cell with the innate-like characteristics of CD8+ T cells in the SARS-CoV-2 infected. In the smoking population, the number of FCGR3A^+^ Cytotoxic CD8 ^+^ T cell subset was significantly elevated, suggesting immune activation and implying a potential association between smoking and the development of vitiligo (64). However, in our patients, this subset was markedly reduced, which may indicate immune dysregulation in individuals with vitiligo.

Upon stimulation with interleukin-4 (IL-4), B cells undergo proliferation and regulate immunoglobulin class switching (such as IgG1 and IgE), thereby facilitating T-cell development, which may contribute to the pathogenesis of vitiligo (65). However, no significant differences in the frequency of B cell subsets were observed in our data. Nevertheless, the role of B cell subpopulation heterogeneity in the pathogenesis and progression of vitiligo remains inadequately explored. In this study, we identified a subpopulation within memory B cells characterized by high EGR1 expression, which we defined as the EGR1+ memory B cell subpopulation. This subpopulation is enriched in pathways related to the cellular response to calcium ions and the regulation of hemopoiesis. GO analysis revealed significant enrichment of genes related to osteoclast differentiation and the IL-17 signaling pathway. Several genes that are not fully annotated, such as AC245014.3, SNORD3B-1, and AL021155.2, are also expressed in the EGR1+ memory B cell subpopulation. The expression of these genes may serve as critical factors in promoting B cell activation, thereby mediating the development of vitiligo. EGR1 mediates oxidative phosphorylation metabolic reprogramming in B-cell malignancies, thereby inducing resistance to ibrutinib (66). Studies in mouse models have shown that knockout of EGR1 inhibits the development of normal B cells and affects the differentiation of pro-B cells and immature B cells (67, 68). However, the reduction in EGR1 cell subpopulations and their functional characteristics in patients with vitiligo require further investigation and characterization.

Among the innate immune cell types, monocytes are less studied in vitiligo. A previous study reported that the proportion of CD80+ monocytes was significantly higher in the peripheral blood of vitiligo patients than in that of healthy controls, suggesting that monocytes may play a role in the development of vitiligo (24). In this study, we re-clustered monocytes and identified two subpopulations that were significantly reduced in vitiligo patients compared with normal controls. However, differential gene expression analysis did not distinguish specific subpopulations. Compared with the healthy controls, monocyte subpopulations expressed high levels of HLA-DRA, FOS, and the uncharacterized gene AC253572.2 in vitiligo patients. The literature also reports a significant increase in FOS expression in patients (69). Pathways related to leukocyte cell-cell adhesion and the regulation of cell-cell adhesion were notably active, as were pathways associated with viral infection.

This study has several limitations inherent to its design and implementation. First, the limited sample size of the discovery cohort for single-cell RNA-seq may compromise statistical robustness and limit the generalizability of our findings. Second, technical constraints precluded the complete validation of some transcriptionally defined cell clusters at the protein level, and the key molecular mechanisms we propose remain speculative without direct functional validation. Finally, the conclusion of impaired Treg cell function is primarily inferred from transcriptomic features suggestive of a Th1-like polarization, which requires confirmation through future *in vitro* functional assays.

In summary, our study delineates the intricate transcriptional landscape of immune cells in vitiligo, emphasizing key cell types and pathways that may play a pivotal role in its pathogenesis. Notably, we observed a reduction of KLRC2 expression in transitional T cells within NK cell and NKT cell subsets. These findings offer valuable insights into potential therapeutic targets for vitiligo and advance our understanding of its underlying immune mechanisms. However, further validation through larger sample sizes is required to substantiate our results.

## Data Availability

The original data presented in this study are openly available in the Genome Sequence Archive (GSA) for Human at the National Genomics Data Center (NGDC), China National Center for Bioinformation. The data can be accessed via the following URL: https://ngdc.cncb.ac.cn/gsa-human/ under the accession number HRA010691. All analysis code is provided in Data Sheet 2.

## Supplementary material

Supplementary Figure 1 UMAP visualization was employed to analyze and compare the overall cellular distributions, as well as the specific patterns of NK cells, NKT cells, B cells, monocytes, and T cells, between vitiligo patients and healthy controls. **(A)** UMAP visualization showing the overall distribution of total cells from each individual sample. **(B)** Distribution of NK and NKT cells across different individuals. **(C)** Distribution of T cell populations across different individuals. Each color represents a distinct cell cluster annotated based on transcriptional signatures. **(D)** Distribution of B cell populations across different individuals. **(E)** Distribution of monocyte populations across different individuals.

Supplementary Figure 2 UMAP visualization depicting the expression distribution and inter-sample heterogeneity of the filtered marker genes **(A-D)** within their corresponding immune cell subsets. **(A)** Expression of KLRC2, a marker for cytotoxic NK cells, within the NK cell subset. **(B)** Expression of FCGR3A (encoding CD16) within the CD8+ T cell subset. **(C)** Expression of STAM within the regulatory T cell (Treg) subset. **(D)** Expression of the early growth response gene EGR1 within the naïve B cell subset. All panels illustrate the distribution of gene expression levels across different samples.

Supplementary Figure 3 KLRC2 Expression in Peripheral Blood Immune Subsets and Total Leukocytes. **(A)** Gating strategy for flow cytometric analysis of KLRC2 expression in peripheral blood immune cell subsets, including monocytes, T cells, NK cells, B cells, and NKT cells. **(B)** Bar graph depicting KLRC2 expression in immune cell subsets of normal controls (HCs) and vitiligo patients, with the x-axis representing specific cell subsets and the y-axis indicating the proportion of KLRC2 expression in these subsets. **(C)** Heatmap of gene expression from bulk RNA sequencing of peripheral blood samples from vitiligo patients and normal controls. **(D)** Bar graph depicting the expression of KLRC2 at the bulk transcriptional level. Asterisks denote statistical significance, with * indicating *P*<0.05 and ** indicating *P*<0.01.
