# Supplementary figures and images for: Single-cell transcriptomics reveals systemic immune dysregulation in non-segmental vitiligo
Source: Front Immunol. 2025 Dec 8;16:1698566. doi: 10.3389/fimmu.2025.1698566 (PMC12719421; doi:10.3389/fimmu.2025.1698566)

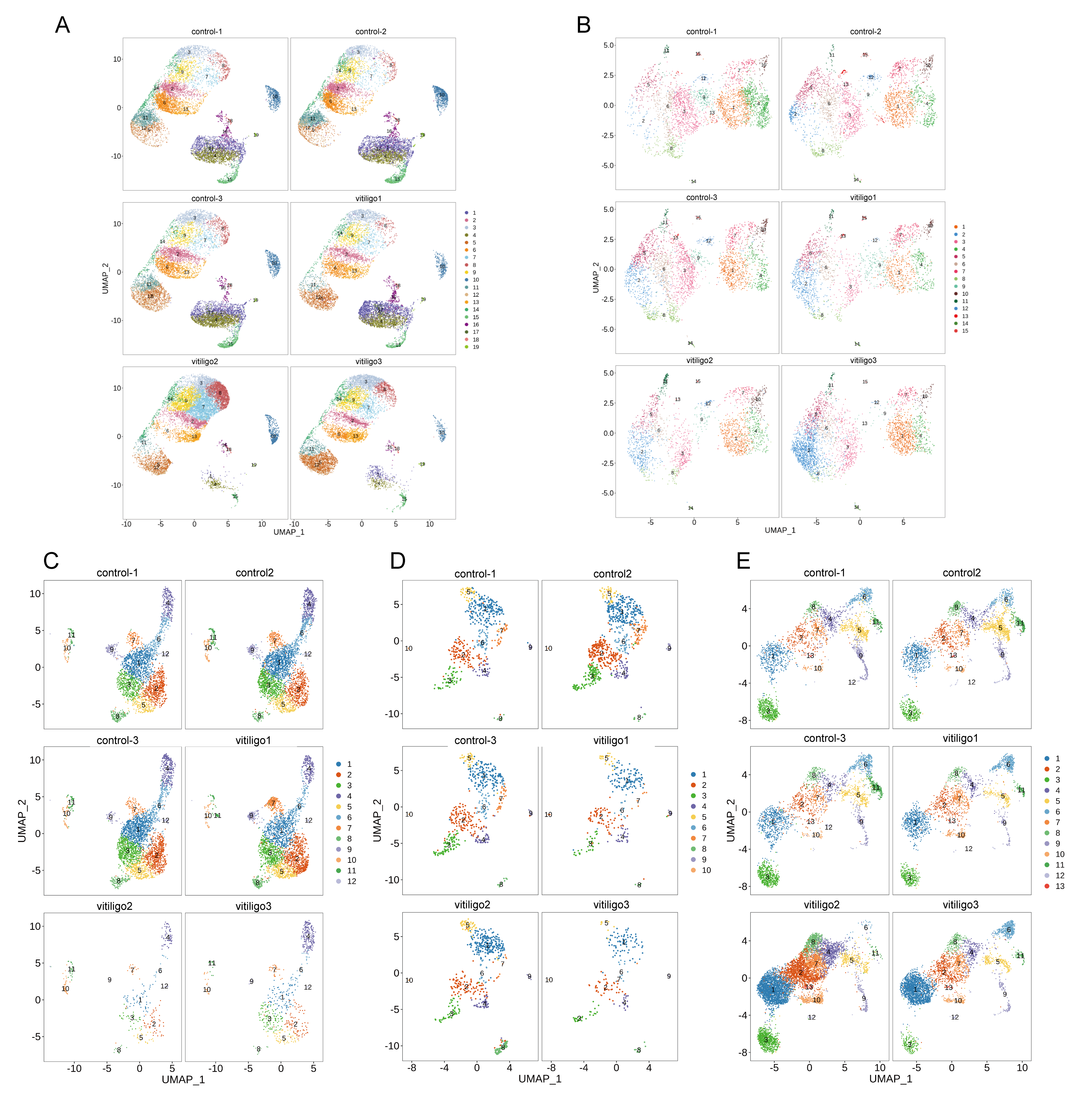

Supplement: Supplementary Figure 1 — UMAP visualization was employed to analyze and compare the overall cellular distributions, as well as the specific patterns of NK cells, NKT cells, B cells, monocytes, and T cells, between vitiligo patients and healthy controls. (A) UMAP visualization showing the overall distribution of total cells from each individual sample. (B) Distribution of NK and NKT cells across different individuals. (C) Distribution of T cell populations across different individuals. Each color represents a distinct cell cluster annotated based on transcriptional signatures. (D) Distribution of B cell populations across different individuals. (E) Distribution of monocyte populations across different individuals. [file Image1.tif]

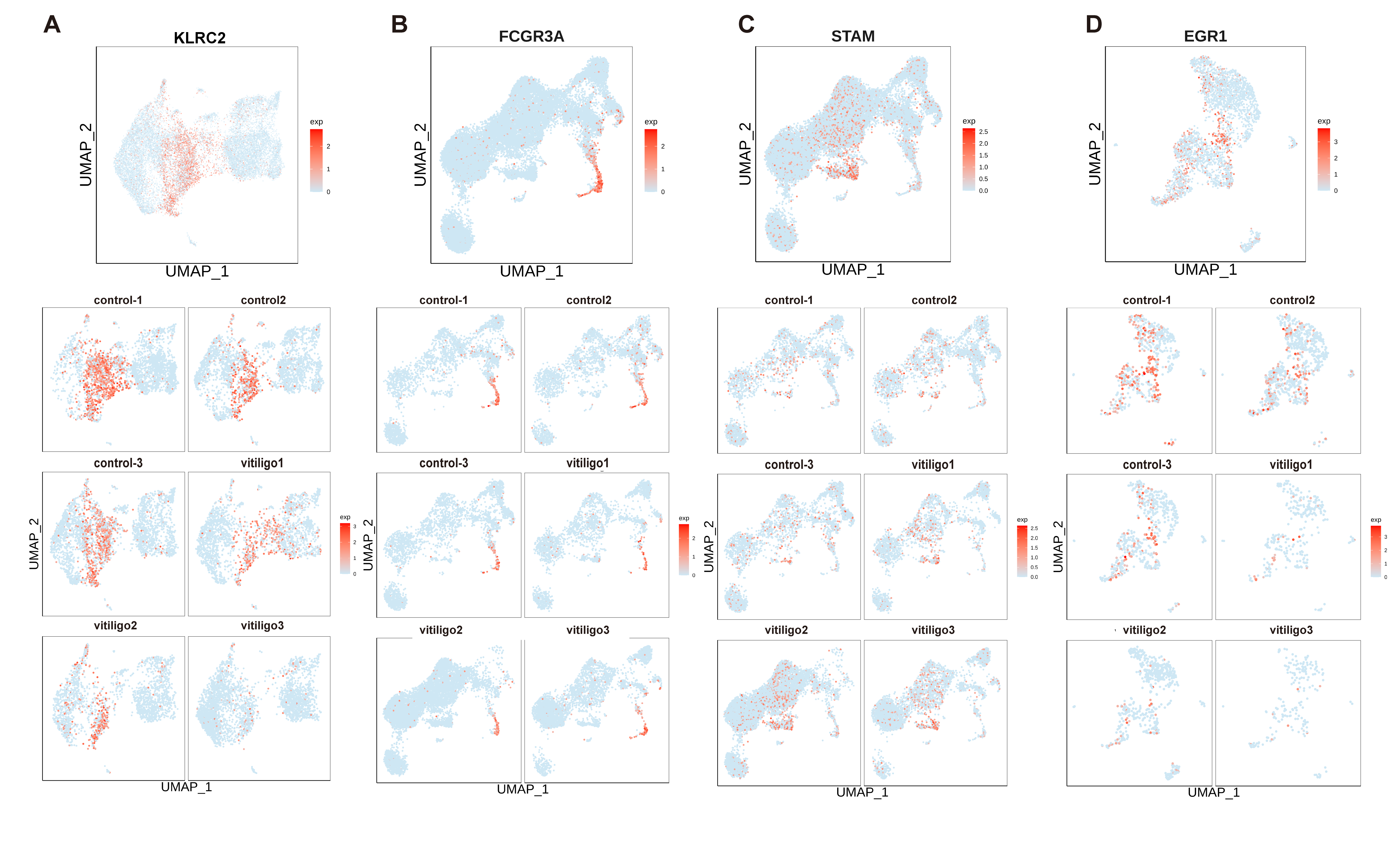

Supplement: Supplementary Figure 2 — UMAP visualization depicting the expression distribution and inter-sample heterogeneity of the filtered marker genes (A-D) within their corresponding immune cell subsets. (A) Expression of KLRC2, a marker for cytotoxic NK cells, within the NK cell subset. (B) Expression of FCGR3A (encoding CD16) within the CD8+ T cell subset. (C) Expression of STAM within the regulatory T cell (Treg) subset. (D) Expression of the early growth response gene EGR1 within the naïve B cell subset. All panels illustrate the distribution of gene expression levels across different samples. [file Image2.tif]

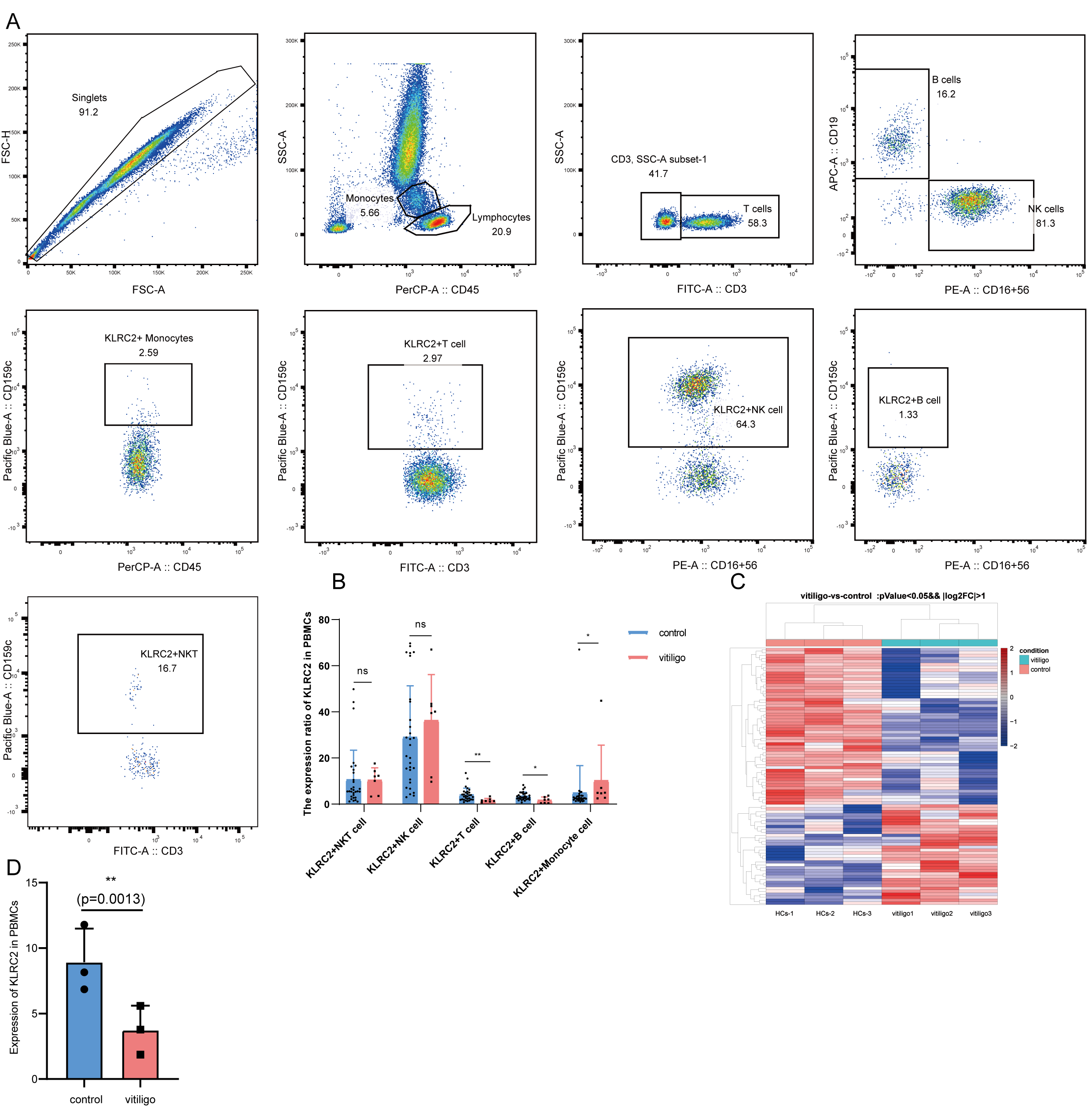

Supplement: Supplementary Figure 3 — KLRC2 Expression in Peripheral Blood Immune Subsets and Total Leukocytes. (A) Gating strategy for flow cytometric analysis of KLRC2 expression in peripheral blood immune cell subsets, including monocytes, T cells, NK cells, B cells, and NKT cells. (B) Bar graph depicting KLRC2 expression in immune cell subsets of normal controls (HCs) and vitiligo patients, with the x-axis representing specific cell subsets and the y-axis indicating the proportion of KLRC2 expression in these subsets. (C) Heatmap of gene expression from bulk RNA sequencing of peripheral blood samples from vitiligo patients and normal controls. (D) Bar graph depicting the expression of KLRC2 at the bulk transcriptional level. Asterisks denote statistical significance, with * indicating P<0.05 and ** indicating P<0.01. [file Image3.tif]
